# OVA66, a Tumor Associated Protein, Induces Oncogenic Transformation of NIH3T3 Cells

**DOI:** 10.1371/journal.pone.0085705

**Published:** 2014-03-14

**Authors:** Wei Rao, Guohua Xie, Yong Zhang, Shujun Wang, Ying Wang, Huizhen Zhang, Feifei Song, Renfeng Zhang, Qinqin Yin, Lisong Shen, Hailiang Ge

**Affiliations:** 1 Shanghai Institute of Immunology, Shanghai Jiaotong University (SJTU) School of Medicine, Shanghai, China; 2 Department of Clinical Laboratory, Xinhua Hospital, Shanghai Jiaotong University (SJTU) School of Medicine, Shanghai, China; National Cancer Institute, NIH, United States of America

## Abstract

The tumor associated antigen OVA66 has been demonstrated to be highly expressed in malignant tumors and implicated in various cellular processes. To further elucidate its oncogenic character, we established an OVA66 stably overexpressed NIH3T3 cell line and a vector transfected control, named NIH3T3-flagOVA66 and NIH3T3-mock, respectively. NIH3T3-flagOVA66 cells showed faster cell cycling, proliferation, cell migration and more resistance to 5-fluorouracil-induced apoptosis. When NIH3T3-flagOVA66 and NIH3T3-mock cells were injected into nude mice for xenograft tumorigenicity assays, the NIH3T3-flagOVA66 cells formed tumors whereas no tumors were observed in mice inoculated with NIH3T3-mock cells. Analysis of PI3K/AKT and ERK1/2 MAPK signaling pathways by serum stimulation indicated hyperactivation of AKT and ERK1/2 in NIH3T3-flagOVA66 cells compared with NIH3T3-mock cells, while a decreased level of p-AKT and p-ERK1/2 were observed in OVA66 knocked down HeLa cells. To further validate if the p-AKT or p-ERK1/2 is essential for OVA66 induced oncogenic transformation, we treated the cells with the PI3K/AKT specific inhibitor LY294002 and the ERK1/2 MAPK specific inhibitor PD98059 and found either inhibitor can attenuate the cell colony forming ability in soft agar and the cell viability of NIH3T3-flagOVA66 cells, suggesting aberrantly activated AKT and ERK1/2 signaling be indispensible of the tumorigenic role of OVA66. Our results indicate that OVA66 is important in oncogenic transformation, promoting proliferation, cell migration and reducing apoptosis via hyperactivating PI3K/AKT and ERK1/2 MAPK signaling pathway. Thus, OVA66 might be a novel target for early detection, prevention and treatment of tumors in the future.

## Introduction

The cancer/testis antigens, known as an important group of proteins that are predominantly expressed in testis but aberrantly activated or expressed in various types of human cancer, are potentially critical immunotherapeutic targets, and possible biomarkers for early diagnosis and prognosis of human cancer [1]. Serological analysis of recombinant cDNA expression libraries (SEREX) which is based on immunoscreening of tumor cDNA expression libraries with sera from the autologous patients is broadly applicable to identification and analysis of cancer antigens [2]. In our previous study, a novel tumor-associated antigen, ovarian associated antigen 66 (OVA66), was first identified by SEREX of an ovarian carcinoma cDNA expression library [3], [4]. It is precisely identical to the *CML66* gene which was initially identified in a chronic myelogenous leukemia (*CML*) cDNA expression library [5]. Our studies on OVA66 expression in human tumor tissues showed remarkably increased expression in most human tumor tissues [6]. We also identified a HLA-A2-restrictive cytotoxic T lymphocyte epitope of OVA66 (CML66) that induced IFN-γ producing T cells [3], [4]. Our previous study also showed that knocking down *OVA66* gene in HeLa cells retarded cell proliferation and promoted apoptosis both *in vitro* and *in vivo* [7]. Besides, we also found cell growth and motility was significantly enhanced by *in vitro* assays after introducing the *OVA66* gene into hepatocellular carcinoma smmc-7721 cells [8]. However, identifying the exact role of OVA66 in tumorigenesis and cancer development requires more investigations.

In our recent studies of OVA66, a recombined eukaryotic expression vector pFlag-OVA66 and an empty vector was transfected into normal mouse fibroblast cell line NIH3T3. The stably transfected NIH3T3 cell clones were isolated, and designated as NIH3T3-flagOVA66 and NIH3T3-mock cells, respectively. Cell cycle analysis, MTT proliferation assay and plate colony formation assay indicated that OVA66 overexpression in NIH3T3 cells promoted cell cycling and proliferation remarkably. The monolayer wound healing and transwell migration assays showed OVA66 improved the cell migrative potential. In addition, NIH3T3-flagOVA66 cells were also more resistant to 5-fluorouracil (5-FU) induced apoptosis compared with NIH3T3-mock cells. *In vivo* experiments showed that the nude mice xenografted with NIH3T3-flagOVA66 cells could form tumors, although they needed more time and formed smaller solid tumors than that xengrafted with typical HeLa cells which endogenously expressed high level of OVA66; whereas no tumors were observed in nude mice injected with NIH3T3-mock cells.

We subsequently showed that NIH3T3-flagOVA66 cells had significantly higher serum-stimulated phosphorylation of AKT and ERK1/2 compared with NIH3T3-mock cells, indicating that oncogenic transformation of OVA66 overexpressing NIH3T3 cells resulted from hyperactivation of the PI3K/AKT and ERK1/2 MAPK signaling pathways. Either blocking the PI3K/AKT signaling by LY294002 or ERK1/2 MAPK signaling by PD98059 abolished the OVA66 promoted cell proliferation and colony formation capacities in soft agar, although inhibiting ERK1/2 MAPK signaling showed less effect on OVA66 regulated cell migration, suggesting a different role of the two signaling pathways in the process of OVA66 induced tumorigenesis.

In conclusion, our results provide the evidences that *OVA66* stably transfected NIH3T3 cells can malignantly transform into tumor cells, and manifest several tumorigenic characteristics both *in vitro* and *in vivo*.

## Methods

### Cell culture

NIH3T3 cells were obtained from the Shanghai Institutes for Biological Sciences, Chinese Academy of Sciences, grown in DMEM (Invitrogen) supplemented with 10% FBS (Invitrogen) and maintained in cell-specific media at 37°C in a humidified atmosphere containing 5% CO_2_.

### Plasmid construction, transfection and generation of stable NIH3T3-flag-ova66 cell line

pFlag-CMV-4 vector is laboratory stock. The OVA66 gene was made by PCR amplification of HeLa cDNA and subcloned into pGEM-T vector. An EcoRI- and KpnI-restricted fragment of OVA66 gene was finally ligated into pFlag-CMV-4 expression vector.

Before transfection, NIH3T3 cells were plated into 6-well plates at 2×10^5^ cells/well. For transfection, 8 ug pFlag-CMV-OVA66 expression vector and pFlag-CMV-4 control vector was used with Lipofectamine™ 2000 transfection agent (Invitrogen). After 48 h, transfected NIH3T3 cells were trypsinized and plated into culture dishes, maintained in growth medium containing 600 μg/ml G418 for 2–3 weeks. Individual colonies were then selected and maintained in the presence of 300 μg/ml G418.

### Preparation of OVA66 monoclonal antibodies

**Figure 1:**
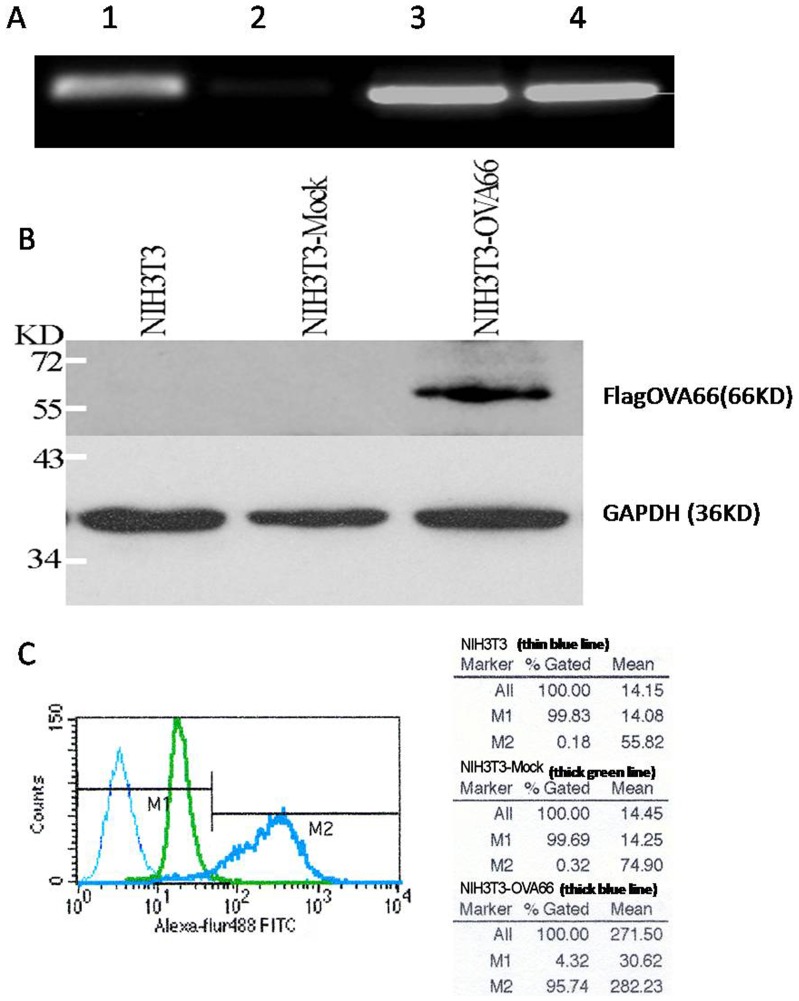
NIH3T3-flagOVA66 cells showed a stably expression of OVA66 mRNA and protein. A. OVA66 mRNA level was examined by RT-PCR. Lane 1 and 2, OVA66 of NIH3T3-flagOVA66 and NIH3T3-mock; Lane 3 and 4, GAPDH of NIH3T3-flagOVA66 and NIH3T3-mock cells. B. Western blot analysis of flagOVA66 protein level in NIH3T3-flagOVA66 cells. C. Quantization of OVA66 protein by FACS analysis using intracellular staining. The thick blue line indicated the expression of OVA66 in NIH3T3-flagOVA66 cells.

Plasmid pET32b-OVA66, encoding an *OVA66*–*His*-tagged fusion gene, was transformed in *Escherichia coli* BL21 (DE3). His-OVA66 recombinant protein was expressed, and purified using Ni^2+^-nitrilotriacetate resin (Machery-Nagel), identified by SDS-PAGE electrophoresis. Antibodies to recombinant OVA66 were raised using His-OVA66 and Freund's complete adjuvant in mice. Subsequently, mouse serum IgG was isolated and purified using Nab protein G spin chromatography kit (Pierce) according to manufacturer’s protocol. The concentration of purified mouse IgG was determined by the BCA method (Pierce) as described in the manufacturer’s protocol. This purified IgG (1 mg/ml concentration), specific to OVA66 and named 4G9 (seen in FigureS1), was used as anti-OVA66 antibody for our experiments as described below.

### Real-time PCR and western blotting

cDNA was synthesized from total RNA extracted from NIH3T3-flagOVA66 and NIH3T3-mock cells. Real-time PCR was performed with a 7500 Fast Real-Time PCR system according to the SYBR Premix Ex Taq (Perfect Real Time) Kit (TaKaRa) instructions, using *β-actin*- and *OVA66* -specific primers: *β-actin*, sense 5′-CTGTCCCTGTATGCCTCTG-3′; antisense, 5′-ATGTCACGCACGATTTCC-3′; *OVA66*, sense 5′-TGCTATTGAGCCTGATGG-3′; antisense 5′-CTGGAAGCCGTATGGTTA-3′. 2^-ΔΔCt^ was calculated as fold change of *OVA66* expression.

NIH3T3-flagOVA66 and NIH3T3-mock cell lysates were extracted using M-PER Mammalian Protein Extraction Reagent (Pierce). Protein concentration was measured using a BCA method with bovine serum albumin (BSA) as the standard. Total cell lysates (30 ug) were separated on 10% SDS-polyacrylamide gels transferred onto PVDF membranes (Bio-Rad) and blocked with TBST supplemented with 5% nonfat milk for 1 hour at room temperature. Membrane was then incubated with rabbit anti-Flag (DYKDDDK) and anti-GAPDH antibodies (Sigma) at 1∶1000 dilution overnight at 4°C. After extensive washing with TBST, membrane was incubated for 1 h with horseradish peroxidase-conjugated goat anti-rabbit IgG (1∶2000) in blocking solution. Blots were detected using ECL Plus Western Blot Detection System (GE).

### Flow cytometry analysis of cell cycle and apoptosis induced by 5-FU

Cell cycle was analyzed by seeding NIH3T3-flagOVA66 and NIH3T3-mock cells at 1×10^6^ cells in a 60-mm dish and allowing the cells to attach for 6 h in growth medium supplemented with 10% FCS. Medium was then changed to growth medium supplemented with 0.5% FCS, maintained for 24 h, and then changed back to growth medium supplemented with 10% FCS for another 24 h. Cells were then trypsinized; cell suspension was prepared in 1.5-ml ice-cold PBS. Absolute ethyl alcohol (2 ml) was added and fixed on ice, followed by washing with PBS, and blocking with 50 μl PBS containing 1% BSA and 0.1% (v/v) Triton X-100 with added RNase (1 mg/ml). Suspension was incubated for 30 min at 37°C before adding 40–50 μl PI (250 μg/ml) away from light for 15 min. Cells were detected in a Becton Dickinson FCM.

NIH3T3-flagOVA66 and NIH3T3-mock cells were plated into 6-well plates and cultivated to 70–80% confluence; 5-FU was added at a concentration gradient of 0–400 μg/ml and maintained for another 24 h or 48 h. Cells were harvested and apoptosis was detected using Annexin V-FITC Apoptosis Detection Kit (BD).

### MTT assay and cloning formation assay

The MTT assay assessed proliferation of NIH3T3-flagOVA66 and NIH3T3-mock cells. Both the NIH3T3-flagOVA66 and NIH3T3-mock cells were plated in a 96-well plate at 5×10^3^ cells per well. Cells were cultivated for 24 h in growth medium supplemented with 0.5% FCS, which was then exchanged with growth medium supplemented with 10% FCS for seven days. Every 24 h, a batch of cells was added with 20 μl MTT solution (5 mg/ml; Sigma) and incubated at 37°C for 4 h. Culture medium was then removed and 200 μl DMSO was added to each well. Crystals of MTT-formazan were dissolved by shaking the plate at room temperature for 10 min. Absorbance was measured on a microplate reader (Bio-Rad) at 570 nm. Cell growth curves were measured according to absorbance of 570 nm on days 1–7. Each group was done in triplicate.

A plate colony formation assay determined the capacity of cells to form colonies. NIH3T3-flagOVA66 and NIH3T3-mock cells were plated in 6-well plates at 0.4×10^3^ cells per well in growth medium supplemented with 10% FCS and incubated at 37°C for 2 weeks. Culture medium was changed every 3–5 days. After 2 weeks, cells were fixed in a phosphate-buffered saline (PBS) solution containing 4% paraformaldehyde, for 30 min on ice and stained with 0.2% crystal violet for 10 min at room temperature. The foci were counted. These experiments were conducted in triplicate in three sets.

### In vitro wound healing assay and cell migration assay

Cells were plated at 2×10^5^ cells/well in 6-well plates, and cultivated in growth medium supplemented with 10% FCS until a monolayer formed. The monolayer was then scratched with sterilized pipette tips and the length of the scratch was recorded under a microscope. After washing with growth medium, cells were cultivated with growth medium supplemented with 10% FCS for 24 h. The length of the scratch was recorded and calculated under a microscope. The relative scratch width means the ration of wound area at 24 hr/wound area at 0 hr. Experiments were conducted in triplicate for three sets.

The cell migration assay was carried out *in vitro* using modified Boyden chambers. A Transwell apparatus (Corning) was separated into upper and lower compartments by polycarbonate filters (8-μm pores). NIH3T3-flagOVA66 and NIH3T3-mock cells (5×10^4^) were separately suspended in 500 μl of growth medium and added into the upper chamber. The lower chamber contained 750 μl growth medium supplemented with 10% FBS. After 24 h incubation at 37°C in a 5% CO_2_ incubator, cells on the upper filter surface were removed by wiping with a cotton swab. Filters were then fixed in 4% paraformaldehyde and stained with crystal violet. Cells that migrated to the lower filter surface were counted in six random fields under a microscope at 200× magnification. Each assay was performed in triplicate.

### Tumor xenograft in nude mice

We used 4–5-week-old BALB/c nude mice (Shanghai SLAC Laboratory Animal Co. Ltd., Shanghai, China) for the *in vivo* tumorigenicity assay of NIH3T3-flagOVA66 and NIH3T3-mock cells. The animal experiment protocol was reviewed and approved by the China Institutional Ethics Review Committee for Animal Experimentation. NIH3T3-flagOVA66 cells, NIH3T3-mock cells and HeLa cells suspended in serum-free DMEM were injected subcutaneously into the right axillary fossa (5×10^6^ cells/animal). Tumors were measured with calipers weekly after tumors appeared, for 5 weeks; volume was calculated by the formula V = 1/2× (length × width^2^). Mice were observed to monitor tumor volume. Mice were then sacrificed; tumors were harvested, partly fixed in 10% buffered formalin, embedded in paraffin, sectioned and stained with H&E. Proteins of tumor tissues formed by NIH3T3-flagOVA66, and tissues where NIH3T3-mock cells were injected, were extracted in T-PER (Thermo) and analyzed by western blotting using OVA66-specific 4G9, anti-phospho-AKT and ERK1/2 antibody.

### Soft agar colony formation assay

Approximately 2.0×10^3^ cells per well were suspended in 0.35% soft agar consisting of complete growth medium containing indicated concentration of DMSO, LY294002 and PD98059 (Selleck, USA) on top of a 0.7% soft agar base layer in 6-well plates. The cells were cultured for more than 2 weeks until visible colonies were formed and then the colonies were photographed and counted. Each assay was performed in triplicate and repeated three times.

### Analysis of serum-stimulated phosphorylation of AKT and ERK1/2

1×10^6^ cells of either NIH3T3-flagOVA66 cells or NIH3T3-mock cells as well as HeLa-NC-shRNA or HeLa-OVA66-shRNA cells were plated in a 6-well plate and allowed to attach in growth medium supplemented with 10% FCS, which was then changed to growth medium with no serum, and maintained for another 24 h. Serum was then added to the medium at concentrations of 10% for indicated times. Besides, if necessary, 10 uM specific inhibitor LY294002, PD98059 (Selleck, USA) or DMSO could be added to the starved cells and maintained for 1 hr before the serum stimulation. Cell lysates were then extracted using M-PER Mammalian Protein Extraction Reagent, containing 1×protease and 1×phosphatase inhibitor cocktail (Pierce). Each sample’s protein concentration was determined by BCA assay. Equal amounts of protein were loaded in 10% SDS-polyacrylamide gels, separated by electrophoresis, and transferred to PVDF membranes (Bio-Rad) and blocked with 5% BSA for 1 h at room temperature. Membranes were then incubated with anti-AKT, anti-phospho-AKT, anti-ERK1/2, and anti-phospho-ERK1/2 (Cell Signaling Technology, USA), anti-ACTIN, GAPDH (Sigma) and 4G9 antibodies at appropriate dilution overnight at 4°C. They were then washed with TBST, incubated with the appropriate secondary antibody (diluted 1∶5000 in blocking buffer) for 1 h at room temperature and washed again. Blotted proteins were detected using ECL Plus Western Blotting Detection reagent (Millipore).

### Statistical analysis

Data reflects at least 3 independent experiments. Data from both *in vitro* and *in vivo* experiments with mice came from groups of 5 mice and were analyzed by Student’s *t*-test. *P*≤0.05 was considered to be statistically significant.

## Results

### Determining flag-OVA66 expression in OVA66 stably transfected NIH3T3 cells

**Figure 2:**
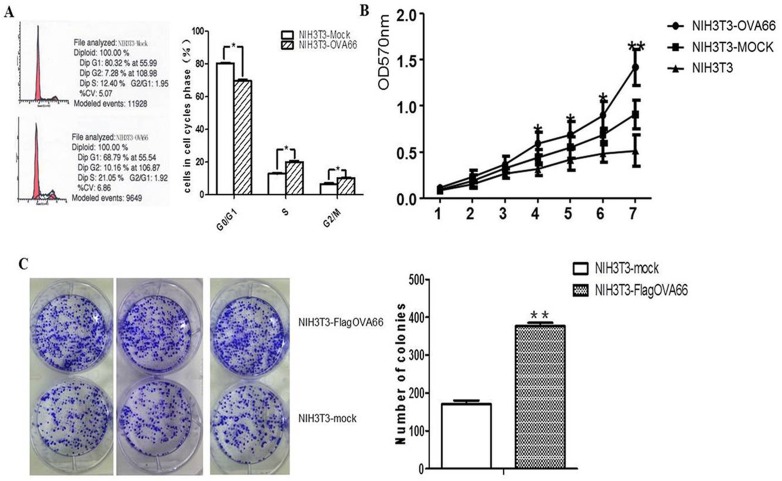
FACS, MTT, and colony-formation assays of NIH3T3-flagOVA66 and NIH3T3-mock cells. A. FACS analysis of cell cycle of the two transfected NIH3T3 cells. OVA66 overexpression in NIH3T3-flagOVA66 cells significantly promote cell cycling compared with NIH3T3-mock cells. B. OVA66 overexpression promoted proliferation of NIH3T3-flagOVA66 cells *in vitro*. Proliferation of NIH3T3 and NIH3T3 transfected with pFlag-OVA66 and pFlag-vector were analyzed by MTT assay for 7 days. C. Colony formation assay in 6-well plates. NIH3T3-flagOVA66 and NIH3T3-mock cells were seeded at 0.4×10^3^ cells per well. After 2 weeks of cultivation, cells were fixed with 4% paraformaldehyde and stained with crystal violet. Numbers of colonies formed by NIH3T3-flagOVA66 and NIH3T3-mock were calculated. The data are represented as mean ± SEM (n = 3), *P≤0.05, **P≤0.01.

OVA66 expression in NIH3T3 cells transfected with pFlag-OVA66 vector or control vector (identified by agarose electrophoresis seen in FigureS2) was firstly examined with real-time PCR, using *β-actin* as internal reference. OVA66 expression in NIH3T3 carrying the pFlag-OVA66 vector was more than 42 times greater than in cells carrying control vector (Table 1). The RT-PCR using GAPDH as the internal control indicated an increased level of OVA66 mRNA in NIH3T3-flagOVA66 cells (Figure 1A). Western blotting also showed OVA66 expressed in NIH3T3-flagOVA66 cell lines while no OVA66 was seen in normal NIH3T3 cells or NIH3T3-mock cells (Figure 1B). In addition, compared to NIH3T3 and NIH3T3-mock cells, the percentage of OVA66 staining was increased by 90% in NIH3T3-flagOVA66 cells using FACS analysis (Figure 1C). The above results demonstrated the over-expression of OVA66 in transfected NIH3T3-flagOVA66 cells.

**Table 1:** Real-time PCR amplification of *OVA66* gene showed OVA66 expression in NIH3T3-flagOVA66 cells increased by greater than 42-fold compared with NIH3T3-mock cells.

	CT value		△CT	△△CT
	β-actin	OVA66	OVA66 - β-actin	
NIH3T3-mock	17.95	28.77	10.82	
NIH3T3-flagOVA66	17.23	22.63	5.4	−5.42
Fold change				2^-△△CT^ = 42.8

### OVA66 overexpression increased S phase percentage and cell proliferation

As OVA66 appears to be an oncoprotein, we then studied changes in transgenic NIH3T3 cells that stably expressed high levels of OVA66. Cell cycle analysis showed the ratio of NIH3T3-flagOVA66 cells in S phase was markedly higher than in control cells (Figure 2A), indicating that OVA66 overexpression in NIH3T3 cells promotes S-phase DNA synthesis and accelerates cell proliferation.

An MTT assay of normal NIH3T3, NIH3T3-mock and NIH3T3-flagOVA66 cells was then carried out. Growth of NIH3T3-flagOVA66 cells increased at days 4–7, compared with NIH3T3-mock and NIH3T3 cells, as calculated by absorbance at 570 nm (Figure 2B). A colony formation assay in 6-well plates was also used to determine cell growth of NIH3T3-flagOVA66 and NIH3T3-mock cells. The data showed the colonies formed per well by NIH3T3-flagOVA66 cells (376.7±8.667) were approximately 55% more than NIH3T3-mock cells (170.7±9.33) (Figure 2C). These results suggest that OVA66 overexpression in NIH3T3 cells increases cell cycle rate and proliferation.

### OVA66 overexpression improves migration ability of NIH3T3-flagOVA66 cells

**Figure 3:**
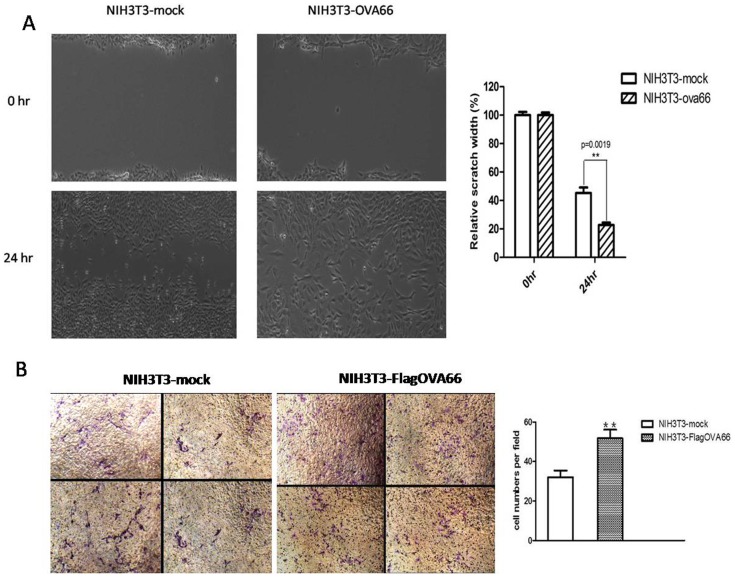
Cell migration assay. A. Assay of NIH3T3-mock and NIH3T3-flagOVA66 cell migration capacity by monolayer wound healing. Magnification: 100×. The relative scratch width(%) means the width at 24 hr/the width at 0 hr. The data represent mean ± SEM (n = 3), **P≤0.01 B. Assay of transfected NIH3T3 cell migration capacity by transwell migration assay *in vitro*. The polycarbonate were stained with Crystal violet and inspected under a microscope at 200×. The Column means the number of cells that migrated the Transwell membranes, the data are represented as mean ± SEM, **P≤0.01.

Our migration assay of NIH3T3-flagOVA66 cells used a wound healing technique and modified Boyden chambers. OVA66-transfected NIH3T3 cells showed enhanced migration capacity compared with NIH3T3-mock cells (Figure 3). In the wound healing assay, 24 h after the scratch was made, the NIH3T3-flagOVA66 cells showed greater migration across the scratch area (the relative scratch width was 22.73%±1.732%) than that observed from the NIH3T3-mock cells (the relative scratch width was 45.24%±3.905%) (P<0.05, Figure 3A). In the Transwell assay, many more NIH3T3-flagOVA66 cells invaded the bottom chamber (51.8±4.3 per field) than NIH3T3-mock cells (32.0±3.4 per field) (*P*<0.01; Figure 3B). Micrographs (200×) were taken and inspected. These results suggest that OVA66 overexpression significantly improves cell migration capacity.

### NIH3T3-flagOVA66 cells were more resistant to 5-fluorouracil induced apoptosis than NIH3T3-mock cells

**Figure 4:**
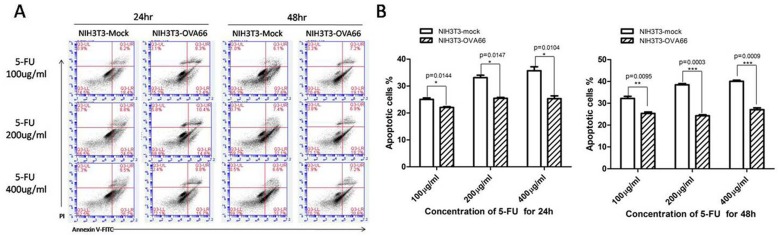
Apoptosis assay. A. FACS analysis of apoptosis using Annexin-V/PI stains detected by BD Accuri C6 flow cytometer. B. Apoptotic cells at 24 h and 48 h after treatment with different concentrations of 5-FU were quantified. The data are represented as mean ± SEM (n = 3), *P≤0.05, **P≤0.01, ***P≤0.001.

5-FU is an anti-metabolic cancer drug [8]. Apoptosis induced by different concentrations of 5-FU was detected in NIH3T3-flagOva66 cells and NIH3T3-mock cells after 24 h or 48 h stimulation. Apoptosis in NIH3T3-mock cells increased as both 5-FU concentration and stimulation time increased, whereas no significant increase was observed in NIH3T3-flagOVA66 cells as 5-FU concentration increased, although apoptosis was increased after 48 h stimulation of 5-FU compared with 24 h stimulation (Figure 4A, B). Totally, 5-FU-induced apoptosis percentage for NIH3T3-flagOVA66 cells was significantly lower than for NIH3T3-mock cells, suggesting that OVA66 overexpression induces apoptosis resistance in NIH3T3 cells.

### Xenografts with NIH3T3-flagOVA66 cells, but not NIH3T3-mock cells, formed tumors in nude mice

**Figure 5:**
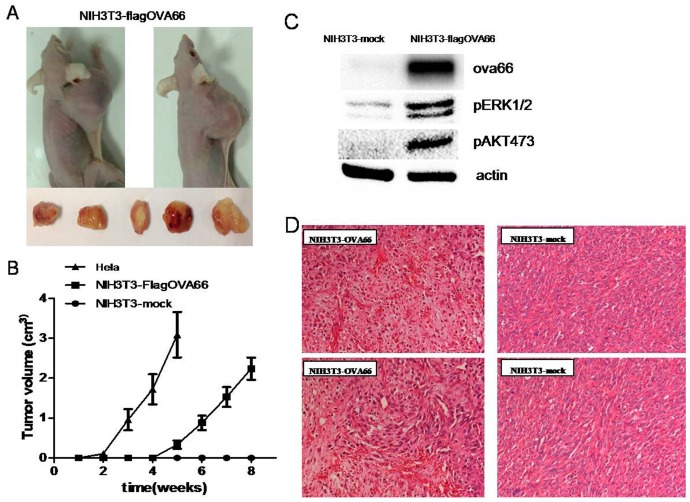
Tumor formation. A. Overall view of tumors formed by NIH3T3-flagOVA66. Tumors in nude mice were seen after injection with NIH3T3-flagOVA66, or with typical OVA66 highly-expressed HeLa tumor cells, whereas none were seen after injection with NIH3T3-mock cells. B. Tumor growth curves for nude mice of different experimental groups (n = 3) C. Western blotting of total proteins extracted from tumors formed by NIH3T3-flagOVA66 and tissues where NIH3T3-mock cells were injected, using actin as a loading control. D. HE staining of tissues from mice inoculated with NIH3T3-flagOVA66 and NIH3T3-mock, respectively (200×).

*Our in vitro* results indicated that antigen OVA66 can transform normal mouse NIH3T3 fibroblasts into malignant cells. To confirm its tumorigenic potential, we performed *in vivo* studies of tumor xenografts in nude mice, using NIH3T3-mock as the negative control and HeLa cells as the positive control. NIH3T3-flagOVA66, NIH3T3-mock or HeLa cells were injected subcutaneously into the right axillary fossa (5×10^6^ cells/animal). Three weeks after injecting NIH3T3-flagOVA66 cells into nude mice, nodular neoplasms could be observed while tumors were obvious at 5 weeks. Both NIH3T3-flagOVA66 and HeLa cells developed tumors, although tumors formed by HeLa cells formed earlier and grew faster, whereas the NIH3T3-mock cells failed to form any tumors (Figure 5). Tumors formed by NIH3T3-flagOVA66 or HeLa cells were observed and their volumes and growth curves calculated for 5 weeks after the tumors could be observed (Figure 5B). These data further indicate that tumor antigen OVA66 overexpression in normal mouse NIH3T3 fibroblast leads to oncogenic transformation and is obviously tumorigenic *in vivo*.

Histopathologic examinations showed that much more anaplastic tumor cells had infiltrated locally in tumor tissues of the NIH3T3-flagOVA66 inoculated group while the NIH3T3-mock group, which failed to form tumors, appeared normal (Figure 5D). Total proteins of NIH3T3-flagOVA66 tumor tissues, and corresponding tissues where NIH3T3-mock cells were injected were extracted and then analyzed by western blotting using 4G9, phospho-AKT and phosphor-ERK1/2 antibodies to detect OVA66 expression and phosphorylation of Akt and Erk1/2 (Figure 5C), indicating OVA66 overexpression (confirmed by IHC in Figure S3) in NIH3T3-flagOVA66 formed tumors and aberrantly activated AKT and ERK1/2.

### Enhanced phosphorylation of Akt or Erk1/2 is essential for OVA66 induced oncogenic transformation

**Figure 6:**
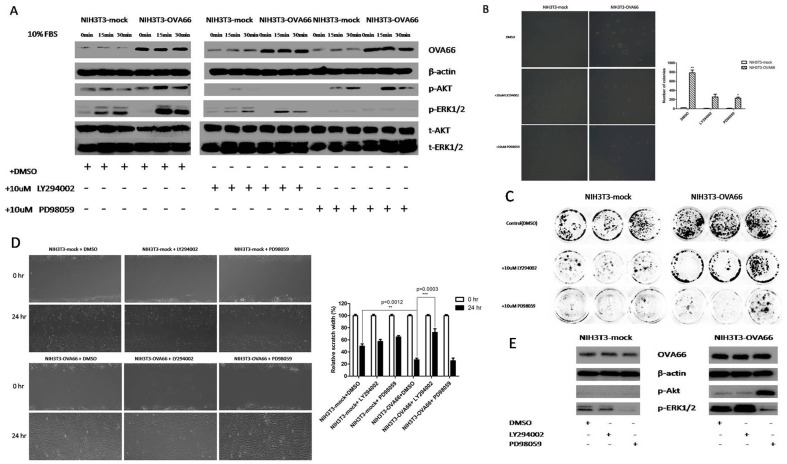
AKT and ERK1/2 phosphorylation. A. Analysis of serum-stimulated phosphorylation of AKT and ERK1/2 as well as total AKT and ERK1/2 protein levels in NIH3T3-flagOVA66 and NIH3T3-mock cells pretreated with control vehicle DMSO, 10 μM LY294002 and 10 μM PD98059 were analyzed by western blotting using actin as a loading control. B. Detection of colony formation in soft agar seeded with NIH3T3-mock and NIH3T3-flagOVA66 cells treated with DMSO, 10 μM LY294002 and 10 μM PD98059. Magnification: 100×, the histogram shows quantification of cell colonies in soft agar. The data are represented as mean ± SEM (n = 3), *P≤0.05, **P≤0.01. C. 400 cells per well were seeded in a 6-well plate for 24 h, followed by treatment with DMSO, 10 μM LY294002 or 10 μM PD98059 for 10 days. Cells were then stained with crystal violet for imaging cell clones in plates. D. Representative wound healing assay images at 0 and 24 hours and the quantification of the relative scratch width after 24 h. Magnification: 100×. Data are mean ± SEM. **P≤0.01, ***P≤0.001. E. Cells of each group of the wound healing assay after 24 h were lysed and the total proteins were analyzed by western blotting.

To determine the effect of OVA66 expression in NIH3T3 cells on phosphorylation of two signal transduction molecules, Akt and ERK1/2, the two stably transfected NIH3T3 cells were serum-starved for 24 h, then pretreated with control vehicle (DMSO), PI3K/AKT specific inhibitor LY294002(10 uM) and ERK1/2 MAPK specific inhibitor PD98059(10 uM) for 1 hr before being incubated with 10%FBS for 0 min, 15 min, 30 min. Cellular proteins were then analyzed by western blotting, using anti-phospho-Akt and anti-phospho-ERK1/2 antibodies. In DMSO pretreated group, phosphorylation of both Akt and Erk1/2 were increased with serum stimulation in NIH3T3-flagOVA66 cells compared to NIH3T3-mock cells (Figure 6A), whereas Akt or Erk1/2 phosphorylation in response to serum stimulation was significantly inhibited by their specific inhibitor in both NIH3T3-mock and NIH3T3-flagOVA66 cells (Figure 6A). Besides, OVA66 knocked down HeLa cells also showed a decreased phosphorylation level of Akt and Erk1/2 under serum stimuli (Figure S4). To further study whether the AKT or ERK1/2 signaling is essential for OVA66 induced transformation, we examined the effect of AKT or ERK1/2 inhibitor on NIH3T3-mock and NIH3T3-flagOVA66 cells with regard to colony forming, cell proliferation and migration ability, respectively. The colony forming capability was analyzed by soft-agar colony-forming assay. When the either specific inhibitor was added to cultures of both cell lines, there was a marked inhibition of colony formation in NIH3T3-flagOVA66 cells (Figure 6B). Note that NIH3T3-mock cells could barely form colonies in soft agar compared to NIH3T3-flagOVA66 cells, suggesting OVA66 induced the transformation required the aberrant activation of AKT or ERK1/2 signaling. The cell proliferation ability was assessed with the plate colony assay and CCK-8 assay, showing that either inhibiting the AKT or ERK1/2 signaling attenuated the cell proliferative potential (Figure 6C and S5). The cell migration ability was assessed with the wound healing assay and the results are displayed in Figure 6D and E. The inhibition of p-AKT by LY294002 clearly resulted in a significant reduction in NIH3T3-flagOVA66 cell migration while p-ERK1/2 inhibition showed less effect (Figure 6D and E), suggesting a different role of the two signaling pathways in regulating the OVA66 induced oncogenic transformation. These results suggest that OVA66 can enhance phosphorylation of Akt and Erk1/2 and probably further affect downstream signal transductions, reversely, the aberrantly activated PI3K/AKT and ERK1/2 MAPK signaling could also be a sine qua non of the oncogenic transformation induced by OVA66. However, the exact tumorigenic mechanisms of OVA66 need our further investigations.

## Discussion

The novel tumor antigen OVA66 was identified from an ovarian cancer cDNA expression library via SEREX. Our previous studies showed that OVA66 was a novel CT antigen that was highly expressed in poorly differentiated, high malignant tumor tissues and cells [3], [6]. Knocking down the *OVA66* gene in HeLa cells significantly inhibited the cell proliferation, migration, metastasis etc [7], considering that tumorigenesis is related in a general sense to imbalanced cell proliferation and death, in which cell growth and apoptosis resistance are favored [10].

Generally, oncogenically transformed normal cells acquire a set of functional capabilities during their development, including self-sufficiency in growth signals, evading apoptosis, cell invasion and metastasis and limitless replicative potential [11]. Some oncogene products have been reportedly to cause oncogenic transformation of normal cells by modulating intracellular signaling pathways that regulate cell growth, migration and apoptosis [12]. In our research, the MTT assay and colony formation assays illustrated the bio-function of OVA66 in cell growth and proliferation. The wound healing and cell migration assays showed enhanced migration capability in NIH3T3-flagOVA66 cells compared with NIH3T3-mock cells. Apoptosis is closely related to tumorigenesis; the subversion of cell death is a principal contributor to the development and progression of cancer [13]. We therefore used different concentrations of 5-FU, the anti-tumor drug [9], on NIH3T3-flagOVA66 cells and NIH3T3-mock cells. More NIH3T3-flagOVA66 cells survived than did NIH3T3-mock cells, suggesting that OVA66 promotes resistance to apoptosis, thus promoting oncogenic transformation of normal NIH3T3 cells. Furthermore, our *in vivo* tumor xenograft assay in nude mice showed that OVA66 overexpressing NIH3T3 cells were malignantly transformed and capable of forming tumors, although their tumorigenic ability *in vivo* was weaker compared with HeLa cells that endogenously expressed a high level of OVA66 protein.

More importantly—as increased activation of MAPK and PI3K pathways can result in malignant transformation [14], [15]—we showed that OVA66 overexpression leads to enhanced phosphorylation of AKT and ERK1/2 when stimulated by serum, which would increase activation of PI3K/AKT and ERK1/2 MAPK pathways. As the ERK1/2 MAPK-mediated signaling pathways regulate cell growth and proliferation and are thus critical to cell cycle regulation [16], OVA66 overexpression in NIH3T3 cells significantly promoted the cell cycle and cell proliferation, probably due to the hyperactivation of the ERK1/2 MAPK pathway. Similarly, Takihara et al. reported that increased MAPK signaling activity was observed in 14-3-3 β transformed cells [17], and other studies have shown14-3-3 proteins play an important role in the PI3K-Akt signaling pathway by interacting with TSC2 protein [18], [19]. In addition, our inhibiting experiments demonstrated that blocking the AKT or ERK1/2 phosphorylation by either specific inhibitor LY294002 or PD98059 could evidently attenuate the OVA66 promoted cell proliferation, migration and abolish oncogenic transformation of NIH3T3 cells to a certain extent, indicating the dysregulation of ERK1/2 MAPK and PI3K/AKT signaling by OVA66 might inversely be essential of tumorigenesis.

In summary, based on our data, we suggest that overexpression of the OVA66 causes oncogenic transformation of NIH3T3 cells via hyper activation of PI3K/AKT and ERK1/2 MAPK signaling pathway. However, a critical question that remains to be elucidated is how the protein OVA66 functioned in different cancer cells. We are currently investigating the oncogenic function and its pivotal role in different cancer cells.

## Supporting Information

Figure S1 **The OVA66 monoclonal antibodies 4G9 was prepared and determined by SDS-PAGE.**
(TIF)

Figure S2 **The recombinant FLAG-OVA66 expression vector and control empty vector was constructed and verified by restriction enzyme cut assay.** (a) Lane 2 and 3, RT-PCR amplificated product of OVA66 gene using the RNA of HeLa cells as the template. (b) Lane2, pFLAG-CMV4-mock plasmid. Lane3, pFLAG-OVA66 plasmid. Lane4, FLAG-OVA66 recombinant plasmid digested by *EcoR I* and *Kpn I*.
(TIF)

Figure S3 **IHC was carried out to detect the expression of OVA66 in tumor tissues formed by NIH3T3-flagOVA66 cells and NIH3T3-mock cells inoculated tissues using 4G9 as the primary antibody. Magnification: 200×.**
(TIF)

Figure S4 **Three cancer cell lines HeLa-NC-shRNA, HeLa-OVA66-shRNA-1 and -2 cells were deprived of serum for 24 hr ahead of adding 10% FBS for 0 min and 15 min.** Cell lysates were then probed with 4G9, p/T-AKT, p/T-ERK1/2 and GAPDH antibodies by WB.
(TIF)

Figure S5 **Cell proliferation of NIH3T3-mock and NIH3T3-flagOVA66 cells treated with DMSO, LY294002 and PD98059 was detected by CCK-8 analysis every 24 h after cells were seeded in a 96-well plate.** The data are mean ± SEM (n = 4).
(TIF)
