# Supplementary figures and images for: OVA66, a Tumor Associated Protein, Induces Oncogenic Transformation of NIH3T3 Cells
Source: PLoS One. 2014 Mar 14;9(3):e85705. doi: 10.1371/journal.pone.0085705 (PMC3954546; doi:10.1371/journal.pone.0085705)

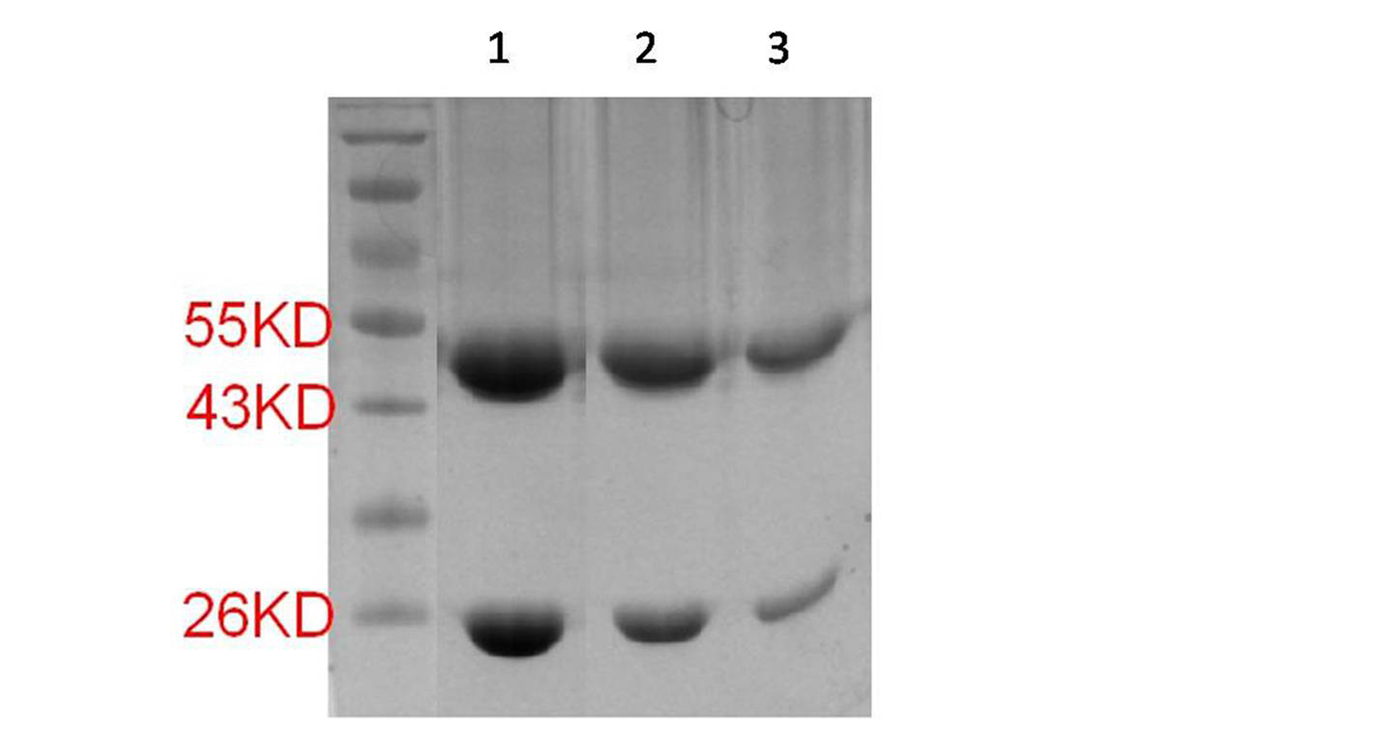

Supplement: Figure S1 — The OVA66 monoclonal antibodies 4G9 was prepared and determined by SDS-PAGE. (TIF) [file pone.0085705.s001.tif]

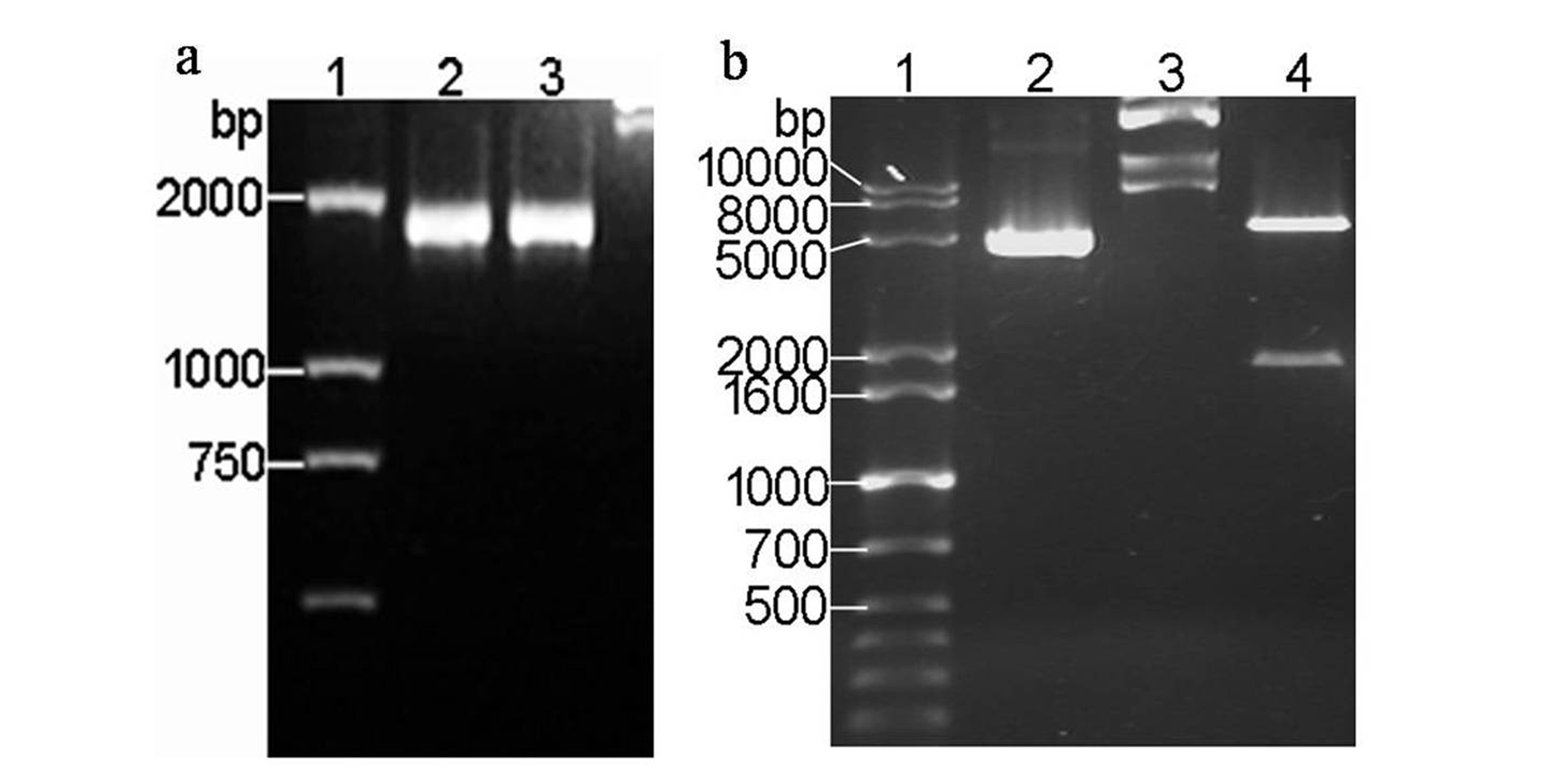

Supplement: Figure S2 — The recombinant FLAG-OVA66 expression vector and control empty vector was constructed and verified by restriction enzyme cut assay. (a) Lane 2 and 3, RT-PCR amplificated product of OVA66 gene using the RNA of HeLa cells as the template. (b) Lane2, pFLAG-CMV4-mock plasmid. Lane3, pFLAG-OVA66 plasmid. Lane4, FLAG-OVA66 recombinant plasmid digested by EcoR I and Kpn I. (TIF) [file pone.0085705.s002.tif]

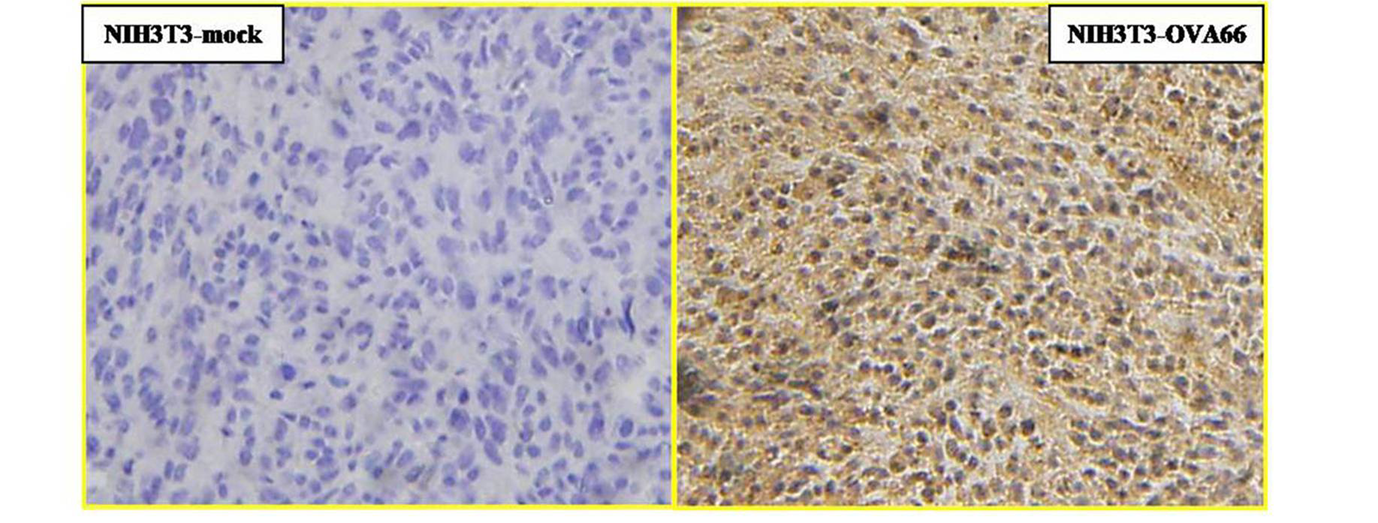

Supplement: Figure S3 — IHC was carried out to detect the expression of OVA66 in tumor tissues formed by NIH3T3-flagOVA66 cells and NIH3T3-mock cells inoculated tissues using 4G9 as the primary antibody. Magnification: 200×. (TIF) [file pone.0085705.s003.tif]

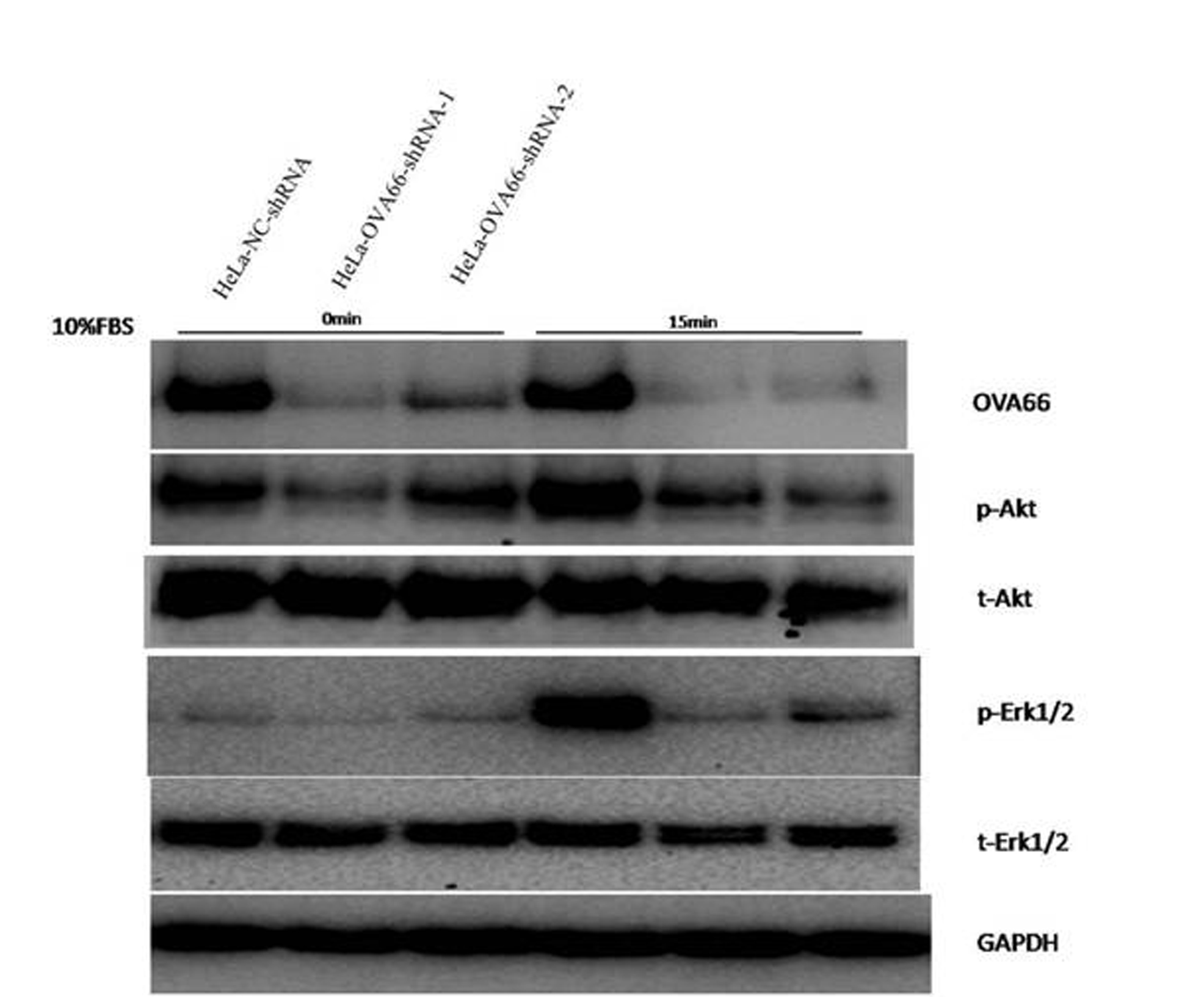

Supplement: Figure S4 — Three cancer cell lines HeLa-NC-shRNA, HeLa-OVA66-shRNA-1 and -2 cells were deprived of serum for 24 hr ahead of adding 10% FBS for 0 min and 15 min. Cell lysates were then probed with 4G9, p/T-AKT, p/T-ERK1/2 and GAPDH antibodies by WB. (TIF) [file pone.0085705.s004.tif]

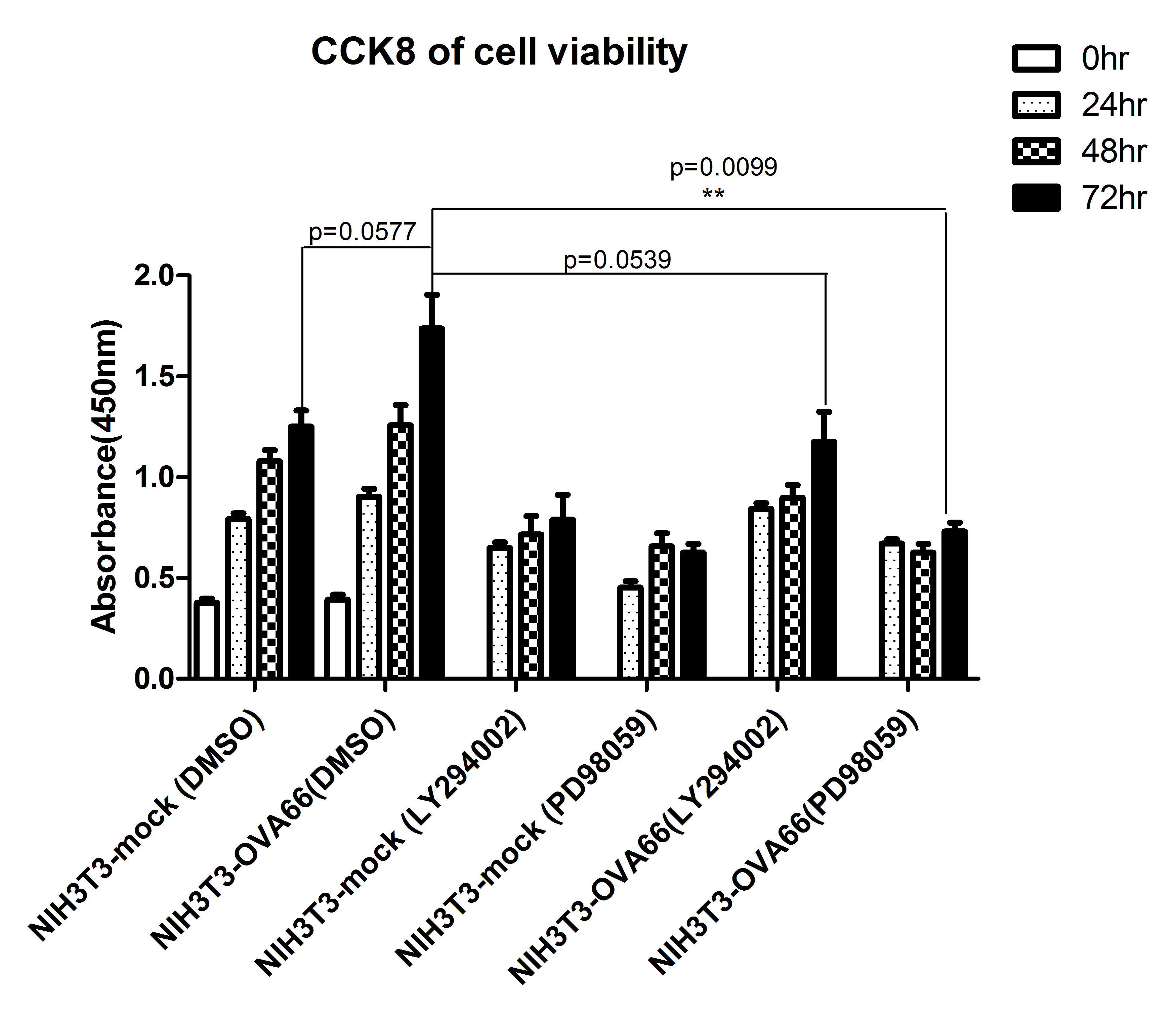

Supplement: Figure S5 — Cell proliferation of NIH3T3-mock and NIH3T3-flagOVA66 cells treated with DMSO, LY294002 and PD98059 was detected by CCK-8 analysis every 24 h after cells were seeded in a 96-well plate. The data are mean ± SEM (n = 4). (TIF) [file pone.0085705.s005.tif]
